# BRCAness and Prognosis in Triple-Negative Breast Cancer Patients Treated with Neoadjuvant Chemotherapy

**DOI:** 10.1371/journal.pone.0165721

**Published:** 2016-12-09

**Authors:** Hirokazu Tanino, Yoshimasa Kosaka, Hiroshi Nishimiya, Youko Tanaka, Naoko Minatani, Mariko Kikuchi, Akiko Shida, Mina Waraya, Hiroshi Katoh, Takumo Enomoto, Norihiko Sengoku, Sabine Kajita, Robert M. Hoffman, Masahiko Watanabe

**Affiliations:** 1 Department of Surgery, Kitasato University School of Medicine, Sagamihara, Kanagawa, Japan; 2 Department of Pathology, Kitasato University School of Medicine, Sagamihara, Kanagawa, Japan; 3 AntiCancer Inc., San Diego, California, United States of America; 4 Department of Surgery, University of California San Diego, San Diego, California, United States of America; University of North Carolina at Chapel Hill School of Medicine, UNITED STATES

## Abstract

BRCAness is defined as the set of traits in which BRCA1 dysfunction, arising from gene mutation, methylation or deletion, results in DNA repair deficiency. In the present study, we addressed BRCAness, therapeutic efficacy, recurrence, and survival in patients with triple negative breast cancer (TNBC) who were treated with neoadjuvant chemotherapy at Kitasato University Hospital, Japan, between April 2006 and October 2012. BRCAness was determined by preoperative core needle biopsy (CNB) specimens and surgical specimens. Assay was performed using Multiplex Ligation-dependent Probe Amplification (MLPA) with P376-B2 BRCA1ness probemix (MRC-Holland, Amsterdam, The Netherlands). The relative copy number ratio of each sample was compared to Human Genomic DNA (Promega, Madison, WI, USA) as reference samples was calculated with Coffalyser.NET default settings. The BRCAness score was calculated with the relative copy number ratio of various DNA sequences. Values of 0.5 or more were determined as the BRCA1-like Type (BRCAness) and those of less than 0.5 as the Sporadic Type to analyze pathological complete response (pCR) rate, recurrence, and survival. pCR (ypT0/Tis/N0) was observed in 15 patients (pCR rate: 37.5%). These patients had no recurrence. Twelve patients recurred, 8 died from breast cancer. The BRCA1-like Type were 22 and Sporadic Type were 18 in CNB specimens. No major differences were observed between the BRCA1-like Type and Sporadic Type with pCR rate, recurrence rate and survival. Twenty four surgical specimens of non-pCR patients were available and 9 were BRCA1-like Type, who had more recurrences (7/9 vs. 5/15), and their relapse-free survival was also lower (p<0.05) than that of Sporadic Type. Seven BRCA1-like Type patients remained BRCA1-like Type in surgical specimens, were worse in recurrence (p<0.01) and survival (p<0.05) compared with 6 patients whose BRCA status in surgical specimens turned to Sporadic Type. New clinical trials assessing the true recurrence (TR) rate of BRCA-type patients are expected since neither platinum-containing drugs nor poly (ADP-ribose) polymerase (PARP) inhibitors are effective against tumors with nonfunctional BRCA genes.

## Introduction

In the late 1990s, breast cancer surpassed stomach cancer to become the most prevalent cancer in Japan, and breast cancer mortality has been increasing ever since [1]. There recently has been progress in personalized medicine, enabling clinicians to select adjuvant therapy based on intrinsic breast cancer subtypes [2]. Patients with increased levels of Ki-67 are known to be associated with poor prognosis [3], and therefore those with high levels of Ki-67 are actively treated with FEC100 and weekly paclitaxel at our institution, the Kitasato University Hospital. This regimen has been administered as a neoadjuvant chemotherapy especially in patients with triple-negative breast cancer (TNBC).

Meanwhile, genetic testing for hereditary breast cancer has become available in Japan, and *BRCA1* and *BRCA2* mutations are relatively frequent in patients who have family history of cancer. It has been suggested that abnormalities in the *BRCA1* gene play an important role in carcinogenesis and in predicting chemotherapy responsiveness in TNBC [4]. BRCA1 and BRCA2 can be inactivated in sporadic cancers as well, which is referred to BRCAness [5]. In BRCA1 mutation carriers, breast tumor samples have a characteristic pattern of DNA gains and losses [6]. An array CGH based classifiers recognizing this genomic pattern of BRCA1-mutated breast tumors was identified [7]. Recently, MLPA assay for the BRCA1ness classification of breast tumors was developed as an alternative for a rather complex array CGH method.

In this study, we sought to determine if BRCAness and response to neoadjuvant chemotherapy could be an effective marker for predicting prognosis in patients with TNBC. Using the MLPA assay, BRCAness was assessed both on core needle biopsy (CNB) specimens obtained prior to neoadjuvant chemotherapy and on surgical specimens. BRCAness recurrence, and survival in patients with TNBC treated with neoadjuvant chemotherapy were correlated. Data of germline BRCA mutations was not available for the patients in this study.

## Patients and Methods

### Patients

A total of 40 patients with TNBC, who were diagnosed and treated with neoadjuvant chemotherapy at Kitasato University Hospital, were enrolled in our study between April 2006 and October 2012. For the neoadjuvant regimen, anthracyclines alone were used in three of these patients, and anthracyclines plus taxanes were used in 37 patients. The median observation period was 46 months (range, 4–85 months). The average age of patients was 53.8 years (range, 26–69 years). Of those, 16 patients (40.0%) were premenopausal. None of the patients were classified as clinical stage 1, 25 patients were classified as stage 2, and 12 patients were classified as stage 3. The efficacy of neoadjuvant chemotherapy was determined in terms of pathological complete response (pCR) rate, which was defined as ypT0/Tis/N0; pCR was observed in 15 patients, and non-pCR was observed in 25 patients (pCR rate, 37.5%). Twelve patients experienced recurrence after surgery, and 8 patients died from the original disease. Twelve patients underwent total mastectomy, while 28 (70.0%) had breast-conserving surgery (Table 1).

**Table 1:** Characteristics of patients.

Factors	N*	%		N*	%
Patients	40	100	Histology		
Ages (range years)	53.8 (26–69)		Invasive Ductal Carcinoma	37	92.5
Menopausal Status			Invasive Lobular Carcinoma	1	2.5
Premenopausal	16	40.0	Special Type	2	5.0
Postmenopausal	24	60.0	Chemotherapy		
Tumor Size**			A + T combined***	37	92.5
cT1	2	5.0	A alone***	3	7.5
cT2	24	60.0	Core Needle Biopsy Specimens		
cT3	8	20.0	BRCA1-like Type	18	45.0
cT4	6	15.0	Sporadic Type	22	55.0
Lymph node status**			Surgical Specimens		
cN0	2	5.0	pCR	15	37.5
cN1	31	77.5	BRCA1-like Type	9	22.5
cN2	5	12.5	Sporadic Type	15	37.5
cN3	2	5.0			

* number of the patients,

**UICC TNM,

***A; Anthracycline, T; Taxan

### DNA isolation and MLPA

CNB specimens and surgical specimens were used for MLPA analysis. Representative hematoxylin and eosin-stained slides from formalin-fixed, paraffin-embedded (FFPE) specimens were reviewed by a pathologist. Tumor tissue was selected and dissected using a scalpel. DNA was isolated from tumor tissue using the QIAamp DNA FFPE Tissue Kit (Qiagen, Hilden, Germany).

Classification of subtypes of BRCAness was performed using MLPA with P376-B2 BRCA1ness probemix (MRC-Holland, Amsterdam, The Netherlands) as previously reported [8]. This probemix covers the chromosomal regions that have been found to be gained in 3q22-29, 6p21-22, 10p14, 12p13, 13q31-34, and lost in 3p21, 5q12-23, 10q23, 12q21-23, 14q22-24 and 15q15-21 translocation in previous studies [9]. MLPA was carried out at FALCO Biosystems Ltd. MLPA was performed according to the manufacturer’s instructions. Briefly, 5 μl of DNA (50–100 ng) was denatured at 98°C for 5 min and subsequently cooled down to 25°C. After adding the probe mix, the sample was denatured at 95°C for 1 min, and the probes were allowed to hybridize at 60°C for 16 h. Probe ligation was performed with the temperature-stable ligase-65 enzyme for 15 min at 54°C. Then the ligase was inactivated by incubation at 98°C for 5 min. PCR was carried out by 35 cycles of 95°C for 30s, 60°C for 30s, and 72°C for 60s followed by 72°C for 20 min. The PCR products were analyzed on a 3130xl genetic analyzer (Life Technologies, Foster City, CA) using Genescan 500 ROX size standard (Life Technologies, Foster City, CA). Data analysis was performed using the Coffalyser.NET software (MRC-Holland, Amsterdam, The Netherlands). The relative copy number ratio of each sample was compared to Human Genomic DNA (Promega, Madison, WI, USA) as reference samples was calculated with Coffalyser.NET default settings. The BRCAness score was calculated with the relative copy number ratio of various DNA sequences. The relative copy number ratio from Coffalyser.NET for all the 38 target specific probes was used for prediction analysis for microarrays (PAM). The training set generated by MRC-Holland with P376-B2 Lot 0911 was used for the PAM. Each sample was analyzed twice, and the average score was used for this analysis. The BRCAness analysis was performed completely blinded to clinical information. The cutoff for BRCAness was 0.5 [9]. This study was performed according to the guidelines of the Declaration of Helsinki, as amended in Edinburgh, Scotland in October 2000. Institutional Review Board approval and written informed consents from the patients were obtained before any specific study procedures were begun. The present study was approved by the Ethics Committee of Kitasato University.

### Data analysis

The specimens obtained by CNB and from surgery were classified as either the BRCA1-like type or the sporadic type. Clinico-pathological factors, clinical efficacy of neoadjuvant chemotherapy, pCR rates, recurrence, and survival were compared between the two types. The T and N factors and the clinical stages of cancer were evaluated in accordance with the Union for International Cancer Control (UICC) version 7.

### Statistical analysis

The statistical difference between BRCA1-like type and sporadic type CNB specimens was assessed using the Chi-square test for clinico-pathological variables. Statistical analysis of BRCA1-like type and sporadic type CNB and surgical specimens was performed using the log-rank test for recurrence and survival rate curves. The SAS software package JMP version 9.0 (SAS Institute, Cary, NC, USA) was used to conduct statistical analyses. A p<0.05 was considered as significant.

## Results

### Association between BRCA1 types of CNB specimens and clinico-pathological factors

The values had a mean of 0.565 with a standard deviation of 0.313 with CNB samples and a mean of 0.434 with a standard deviation of 0.335 on surgical specimens. Of the tumor specimens obtained by CNB, 22 specimens were found to be of the BRCA1-like type and 18 were of the sporadic type. No statistically significant differences were observed between the BRCA1-like type and the sporadic type with regard to the preoperative tumor size, the presence or absence of lymph node metastasis, staging, the pCR rate (8/22, 36.4% vs. 7/18, 38.9%, respectively), or recurrence and survival rates (Table 2).

**Table 2:** BRCAness and Characters.

	BRCA1-like Type		Sporadic Type		p
	(n = 22)		(n = 18)		
Characters	No.	%	No.	%	
Menopausal Status					
Premenopausal	10	45.5	6	33.3	0.44
Postmenopausal	12	54.5	12	66.7	
Tumor Size					
cT1-cT2	15	68.2	11	61.1	0.64
cT3-cT4	7	31.8	7	38.9	
Lymphnode Metastasis					
cN0-cN1	17	77.3	16	88.9	0.33
cN2-cN3	5	22.7	2	11.1	
Surgical Specimens					
pCR	8	36.4	7	38.9	0.86
non pCR	14	63.6	11	61.1	
Recurrence					
negative	15	68.2	13	72.2	0.78
positive	7	31.8	5	27.8	
Prognosis					
Alive	18	81.8	14	77.8	0.75
Died	4	18.2	4	22.2	

### Association between BRCA1 types of surgical specimens and recurrence/survival

**Fig 1:**
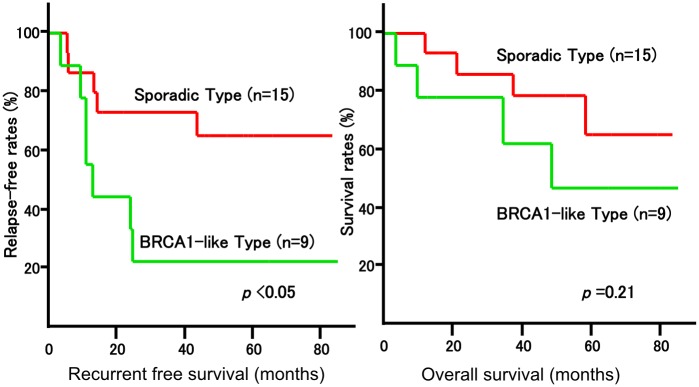
Association between BRCAness of Surgical Specimens and Recurrence/Survival. Among 40 patients, 15 patients achieve to pCR and 1 patient had cancer on only lymph node. Twenty four breast tumors were assessed by MLPA. The recurrent rate of BRCA1-like type was worse than that of sporadic type significantly. But, survival of two groups were similar.

Fifteen patients achieved pCR and 25 did not. In one of 25 patients who did not achieve pCR, residual cancer was present only in the lymph node, thus rendering the measurement of the tumor impossible. As a result, surgical specimens that were measureable were analyzed in 24 patients, in whom 9 tumors were found to be of the BRCA1-like type and 15 were of the sporadic type. The recurrence-free survival rate in the BRCA-like type group was significantly lower (p<0.05) (Fig 1).

### Association between change of BRCAness type and recurrence

**Fig 2:**
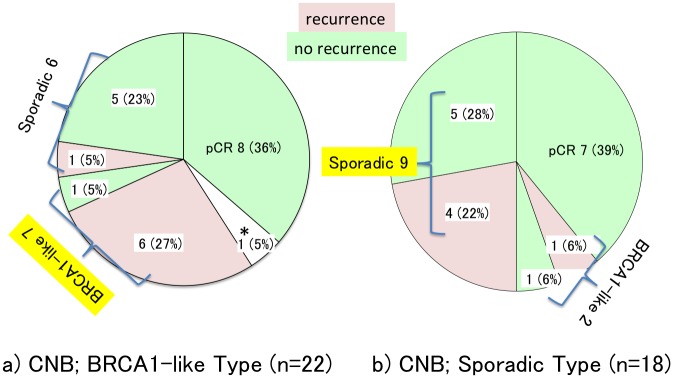
Changes in BRCAness Types and Recurrence. Recurrent patients were shown as pink and green means no recurrence. On 40 of CNB specimens, (a) 22 patients were found to be of BRCA1-like type and (b) 18 were sporadic type. (a) BRCA1-like type; Eight patients (36.4%) achieved pCR. None of pCR patients had a recurrence. Patients whose tumor status was found to remain as BRCA1-like type upon analysis of surgical specimens experienced more recurrences than those who changed to sporadic type. (83.3% vs. 16.7% retrospectively) (p<0.01) * One patient who did not achieve pCR, residual cancer was present only in the lymph node, thus rendering the measurement of the tumor impossible. (b) Sporadic type; Seven patients (38%) achieved pCR. None of pCR patients had a recurrence. Most non-pCR patients remained sporadic type and such patients recurred at 44% (4/9).

Upon analysis of the CNB specimens, 22 patients were found to be of BRCA1-like type, 8 (36%) of whom had pCR. All of pCR patients had no recurrence. Surgical specimens were evaluated in the rest of the 13 patients. Following treatment, the tumor status of 6 of these patients changed to the sporadic type, whereas the tumors of 7 patients remained BRCA1-like. Patients whose tumor status was found to remain as BRCA1-like type upon analysis of surgical specimens experienced more recurrences than those who changed to sporadic type (6/7, 83.3% vs. 1/6, 16.7%) (p<0.01) (Fig 2).

The values of one patient who changed phenotype from BRCA1-like Type (0.648) to Sporadic Type (0.470) is close to the cutoff point, 0.5. Eighteen patients were found to be of BRCA1-like type on CNB specimens, 7 (38%) of whom had pCR. None of pCR patients had a recurrence. Surgical specimens were evaluated in the rest of the 11 patients. Of surgical specimens, 9 patients remained as sporadic type. Among them, 4 patients had a recurrence (Fig 2).

## Discussion

BRCA1 plays an important role in the DNA damage repair pathway. Women deficient in the *BRCA1* gene are frequently found to have TNBC [10]. BRCAness is defined as the set of traits in which BRCA1 dysfunction, arising from gene mutation, methylation or deletion, results in DNA repair deficiency [5]. Tumors with a BRCAness phenotype are thought to be highly sensitive to chemotherapy. In this study, we used the MLPA assay to examine somatic BRCAness status, rather than germline mutation status, and sought to determine whether BRCAness status can serve as a reliable predictor of responsiveness to chemotherapy and patient outcome.

In our study, among the patients with TNBC who were assessed, all who achieved pCR with neoadjuvant chemotherapy survived without relapse. However, analysis of the CNB specimens revealed that the difference in pCR rates were not statistically significant between patients whose tumors were classified as the BRCA1-like type and patients whose tumors were classified as the sporadic type, suggesting that BRCA1 classification may not be feasible in predicting responsiveness for usual chemotherapy using anthracycline and taxan.

Improved prognosis has been observed in patients whose Ki-67 levels decreased in post-chemotherapy surgical specimens when compared to CNB samples obtained prior to chemotherapy [11]. We hypothesized, therefore, that comparing the tumor status prior to and after chemotherapy would enable prognosis prediction, and conducted a comparative study of BRCAness in tumor samples obtained by CNB and surgery. When we examined surgical specimens taken from patients who did not achieve pCR, more patients with the BRCA1-like type tumor relapsed than those with the sporadic type tumor (Fig 1). BRCA1-like type tumor relapsed quickly, and all of the recurrent patients were diagnosed within 2 years. However, survival curves of both groups were similar. BRCA1-like type tumors were thought to be sensitive to chemotherapy even after recurrence. These results suggest that in order to improve the prognosis of TNBC, we have to use more effective drug as neoadjuvant chemotherapy if the tumor was found to be BRCA1-like type on CNB specimens. It is possible that these patients relapsed because their tumors were deficient in BRCA1 and were insensitive to anthracyclines and taxanes. Telli *et al*. [12] previously reported that platinum agents were effective against tumors with a BRCAness profile. Platinum-based therapy may result in better prognosis for patients who are unresponsive to anthracyclines and taxanes. We hope that new clinical trials incorporating translational research that further address BRCAness will be conducted. Poly ADP ribose polymerase (PARP) inhibitors, which inhibit DNA single strand break repair, may also be beneficial for this subset of patients with BRCAness phenotypes.

## References

[1] Ferlay J, Shin HR, Bray F, Forman D, Mathers C, Parkin DM. Estimates of worldwide burden of cancer in 2008: GLOBOCAN 2008. Int J Cancer. 2010;127(12):2893–917. 10.1002/ijc.25516 21351269

[2] Goldhirsch A, Wood WC, Coates AS, Gelber RD, Thurlimann B, Senn HJ. Strategies for subtypes—dealing with the diversity of breast cancer: highlights of the St. Gallen International Expert Consensus on the Primary Therapy of Early Breast Cancer 2011. Annals of oncology: official journal of the European Society for Medical Oncology / ESMO. 2011;22(8):1736–47. 10.1093/annonc/mdr304 PMC3144634 21709140

[3] Nishimiya H, Kosaka Y, Yamashita K, Minatani N, Kikuchi M, Ema A, et al Prognostic significance of Ki-67 in chemotherapy-naive breast cancer patients with 10-year follow-up. Anticancer research. 2014;34(1):259–68. 24403472

[4] Von Minckwitz Gunter E H, Fasching Peter A., Jan Hauke, Andreas Schneeweiss, Christoph Salat, Mahdi Rezai, Blohmer Jens U., Zahm Dirk Michael, Christian Jackisch, Bernd Gerber, Peter Klare, Sherko Kummel, Holger Eidtmann, Stefan Paepke, Valentina Nekljudova, Sibylle Loibl, Michael Untch, Schmutzler Rita K. and GBG and AGO-B Study Groups. Pathological complete response (pCR) rates after carboplatin-containing neoadjuvant chemotherapy in patients with germline BRCA (gBRCA) mutation and triple-negative breast cancer (TNBC): Results from GeparSixto. Journal of Clinical Oncology. 2014;32(No 15_suppl): 1005.

[5] Turner N, Tutt A, Ashworth A. Hallmarks of 'BRCAness' in sporadic cancers. Nature reviews Cancer. 2004;4(10):814–9. 10.1038/nrc1457 15510162

[6] Wessels LF, van Welsem T, Hart AA, van't Veer LJ, Reinders MJ, Nederlof PM. Molecular classification of breast carcinomas by comparative genomic hybridization: a specific somatic genetic profile for BRCA1 tumors. Cancer research. 2002;62(23):7110–7. 12460933

[7] Joosse SA, van Beers EH, Tielen IH, Horlings H, Peterse JL, Hoogerbrugge N, et al Prediction of BRCA1-association in hereditary non-BRCA1/2 breast carcinomas with array-CGH. Breast cancer research and treatment. 2009;116(3):479–89. 10.1007/s10549-008-0117-z 18704682

[8] Oonk AM, van Rijn C, Smits MM, Mulder L, Laddach N, Savola SP, et al Clinical correlates of 'BRCAness' in triple-negative breast cancer of patients receiving adjuvant chemotherapy. Annals of oncology: official journal of the European Society for Medical Oncology / ESMO. 2012;23(9):2301–5. 10.1093/annonc/mdr621 22357256

[9] Lips EH, Laddach N, Savola SP, Vollebergh MA, Oonk AM, Imholz AL, et al Quantitative copy number analysis by Multiplex Ligation-dependent Probe Amplification (MLPA) of BRCA1-associated breast cancer regions identifies BRCAness. Breast cancer research: BCR. 2011;13(5):R107 10.1186/bcr3049 22032731 PMC3262220

[10] Metzger-Filho O, Tutt A, de Azambuja E, Saini KS, Viale G, Loi S, et al Dissecting the heterogeneity of triple-negative breast cancer. Journal of clinical oncology: official journal of the American Society of Clinical Oncology. 2012;30(15):1879–87. 22454417 10.1200/JCO.2011.38.2010

[11] Carey LA, Perou CM, Livasy CA, Dressler LG, Cowan D, Conway K, et al Race, breast cancer subtypes, and survival in the Carolina Breast Cancer Study. Jama. 2006;295(21):2492–502. 10.1001/jama.295.21.2492 16757721

[12] Telli M. Optimizing chemotherapy in triple-negative breast cancer: the role of platinum. American Society of Clinical Oncology educational book / ASCO American Society of Clinical Oncology Meeting. 2014:e37–42. 10.14694/EdBook_AM.2014.34.e37 24857126

